# The Relationship between Controlling Nutritional (CONUT) Score and Clinical Markers among Adults with Hepatitis C Virus Related Liver Cirrhosis

**DOI:** 10.3390/nu10091185

**Published:** 2018-08-29

**Authors:** Hiroki Nishikawa, Kazunori Yoh, Hirayuki Enomoto, Noriko Ishii, Yoshinori Iwata, Ryo Takata, Takashi Nishimura, Nobuhiro Aizawa, Yoshiyuki Sakai, Naoto Ikeda, Kunihiro Hasegawa, Tomoyuki Takashima, Hiroko Iijima, Shuhei Nishiguchi

**Affiliations:** Division of Hepatobiliary and Pancreatic disease, Department of Internal Medicine, Hyogo College of Medicine, Nishinomiya, Hyogo 663-8501, Japan; mm2wintwin@ybb.ne.jp (K.Y.); enomoto@hyo-med.ac.jp (H.E.); ishinori1985@yahoo.co.jp (N.Is.); yo-iwata@hyo-med.ac.jp (Y.I.); chano_chano_rt@yahoo.co.jp (R.T.); tk-nishimura@hyo-med.ac.jp (T.N.); nobu23hiro@yahoo.co.jp (N.A.); sakai429@hyo-med.ac.jp (Y.S.); nikeneko@hyo-med.ac.jp (N.Ik.); hiro.red1230@gmail.com (K.H.); tomo0204@yahoo.co.jp (T.T.); hiroko-i@hyo-med.ac.jp (H.I.); nishiguc@hyo-med.ac.jp (S.N.)

**Keywords:** controlling nutritional status score, liver cirrhosis, hepatitis C virus, malnutrition, predictive marker

## Abstract

Aims: To identify the relationship between the Controlling Nutrition Status (CONUT) score and clinical parameters among adults with hepatitis C virus (HCV)-related liver cirrhosis (LC) (*n* = 264, 141 males and 123 females). Methods: The relationship between the CONUT score and clinical variables such as Child-Pugh classification were investigated. We also examined factors linked to poor nutritional state as determined by CONUT score. Results: According to the CONUT score, normal nutritional state was found in 57 patients, mild malnutrition state in 132, moderate malnutrition state in 68 and severe malnutrition state in 7. The CONUT score ranged from 0 to 9 (median = 2) in Child-Pugh A (*n* = 198), 0 to 10 (median = 6) in Child-Pugh B (*n* = 62) and 6 to 9 (median = 7.5) in Child-Pugh C (*n* = 4) (overall, *p* < 0.00001). Multivariate analysis revealed that FIB-4 index, branched-chain amino acid to tyrosine ratio and extracellular water to total body water ratio in bioimpedance analysis were significant for both CONUT score 2 or more, and 5 or more. FIB-4 index had the highest predictability for both CONUT score 2 or more and 5 or more among three parameters. Conclusion: The CONUT score well reflects liver functional reserve among adults with HCV-related LC. FIB-4 index can be useful for malnutrition.

## 1. Introduction

The liver is the target organ for the metabolism of three major classes of molecules (fat, protein and carbohydrate) and it exerts a unique role in carbohydrate metabolism by preserving glucose concentration levels within the normal range [1,2,3,4,5]. Hepatitis C virus (HCV) is a major cause of chronic hepatitis worldwide and is the leading cause of liver cirrhosis (LC) in Japan [6,7]. LC, which develops over a long period of time due to the chronic inflammation in the liver, is well accepted to be an end-stage form of chronic liver disease and is accompanied by numerous nutrition disorders [8]. Of these, protein-energy malnutrition (PEM) is one of the most common complications seen in LC patients, which is associated with high morbidity and mortality in LC patients [9,10,11,12,13]. Thus, appropriate nutritional evaluation is essential for the management of LC patients.

The most extensively used nutritional screening tool is the Subjective Global Assessment (SGA), however, in LC patients, the SGA has been found to be insufficient for the identification of malnourished patients due to the lack of accuracy and reproducibility of nutritional status [14,15,16]. The Controlling Nutritional Status (CONUT) score is an objective tool that is extensively used to evaluate nutritional status in various diseases [17,18,19,20,21,22,23,24,25,26]. The CONUT score is an index calculated from the three laboratory parameters; serum albumin level, total cholesterol level and peripheral lymphocyte count, which are representative markers of protein synthesis, calorie deficiency, and impaired immune defenses, respectively [23,24]. Because the CONUT score is derived based on the laboratory data using blood samples, clinicians can objectively, simply, and continuously evaluate the nutritional status of the subject [23,24]. The CONUT score was originally proposed as a tool for the early detection of poor nutritional status in hospitalized patients [23]. The significant correlation between the CONUT score and clinical outcomes was seen in investigating patients with conditions such as solid malignancies and heart diseases, and particularly, this screening tool has been considered to be an established assessment model for evaluating nutritional aspects in surgically treated patients [17,18,19,20,21,22,25,26,27].

However, there is currently little data regarding the CONUT score and other clinical parameters, including liver fibrosis markers and body composition data in patients with hepatitis C virus (HCV)-related LC. As mentioned earlier, because HCV is a major cause of chronic hepatitis worldwide and is the leading cause of LC and HCC in our country, these data may provide useful information [6,7]. The current study aimed to identify the relationship between the CONUT score and other clinical parameters such as liver function markers, liver fibrosis markers and body composition data in patients with HCV-related LC.

## 2. Patients and Methods

### 2.1. Study Design and Inclusion Criteria

We retrospectively analyzed a total of 264 patients with HCV-related LC who were admitted to Division of Hepatobiliary and Pancreatic disease, Department of Internal Medicine, Hyogo College of Medicine, Hyogo, Japan between February 2006 and November 2015. The diagnosis of LC was determined based on clinical data, including liver biopsy specimens, laboratory data, clinical characteristics of portal hypertension, and/or radiological imaging such as computed tomography and ultrasonography. All analyzed patients had available data for body composition analysis using bioimpedance analysis (BIA). All analyzed patients had detection of HCV antibody and had no evidence of concurrent hepatitis B virus infection, and no clear evidence of drug-induced or alcoholic liver disease.

### 2.2. Exclusion Criteria

We excluded patients with massive ascites requiring abdominal paracentesis from this study as body composition analyses in BIA can be challenging in LC subjects with severe fluid retention. In other words, body weight, skeletal muscle mass index (SMI) and body mass index in BIA can be overestimated in subjects with massive ascites [13]. HCC patients were also excluded from this analysis. SMI in BIA was defined as “appendicular skeletal muscle mass/(height (m))^2^” [28] Upper-SMI was defined as “skeletal muscle mass of upper extremities/(height (m))^2^”. Lower-SMI was defined as “skeletal muscle mass of lower extremities/(height (m))^2^”.

### 2.3. CONUT Score

As described earlier, the CONUT score is a scoring system based on the calculation from the following three parameters; serum albumin level, total peripheral lymphocyte count and total cholesterol level [23,24]. CONUT scores are summarized in Table 1 [23,24]. According to the CONUT score, patients were classified into four groups: (1) Normal nutritional state (CONUT score 0 or 1); (2) mild malnutrition state (CONUT score 2, 3 or 4); (3) moderate malnutrition state (CONUT score 5, 6, 7 or 8) and (4) severe malnutrition state (CONUT score more than 8).

### 2.4. Our Objectives and Ethical Approval

We aimed to elucidate the relationship between the CONUT score and liver function markers, liver fibrosis markers and body composition data. We also examined factors associated with poor nutritional state as determined by CONUT score using univariate and multivariate analyses. The current study protocol strictly adhered to all regulations of the Declaration of Helsinki, and was approved by the institutional review board of Hyogo College of Medicine, Nishinomiya, Hyogo, Japan (approval no. 2117). We have received written inform consent from all participant patients.

### 2.5. Statistical Analysis

For quantitative variables, the statistical analysis among groups was carried out using Student’s *t* test, Mann-Whitney *U* test, Kruskal-Wallis test, Fisher’s exact test or Spearman’s rank correlation coefficient *r_s_* after assessing the normality of their distribution. Variables with *p* value < 0.05 in the univariate analysis were subjected into the multivariate analysis using the logistic regression analysis. Receiver operating characteristics (ROC) curve analysis and area under the ROC curve (AUC) results were presented along with the corresponding optimal cutoff point that maximized the sum of specificity and sensitivity, sensitivity and specificity. Data are presented as median (range) unless otherwise stated. Statistical significance was set at *p* < 0.05. Statistical analysis was carried out with the JMP 13 (SAS Institute Inc., Cary, NC, USA).

## 3. Results

### 3.1. Patient Characteristics

**Figure 1:**
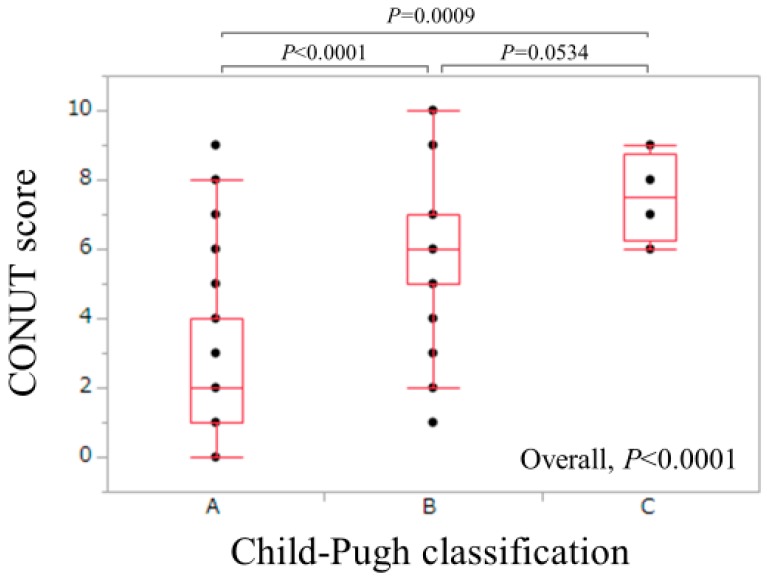
The CONUT score according to Child-Pugh classification. The CONUT score ranged from 0 to 9 (median, 2) in Child-Pugh A (*n* = 198), 0 to 10 (median, 6) in Child-Pugh B (*n* = 62) and 6 to 9 (median, 7.5) in Child-Pugh C (*n* = 4) (*p* values: Child-Pugh A vs. B, *p* < 0.0001; Child-Pugh B vs. C, *p* = 0.0534, Child-Pugh A vs. C, *p* = 0.0009; overall, *p* < 0.00001).

Baseline data in all cases (*n* = 264, 141 males and 123 females) are presented in Table 2. The age ranged from 25.5 to 94.0 years (median, 68.0 years). The CONUT score ranged from 0 to 10 (median, 3). According to the CONUT score, normal nutritional state was found in 57 patients, mild malnutrition state in 132, moderate malnutrition state in 68 and severe malnutrition state in 7. There were 198 patients in Child-Pugh A, 62 in Child-Pugh B and 4 in Child-Pugh C. The CONUT score ranged from 0 to 9 (median, 2) in Child-Pugh A, 0 to 10 (median, 6) in Child-Pugh B and 6 to 9 (median, 7.5) in Child-Pugh C (*p* values: Child-Pugh A vs. B, *p* < 0.0001; Child-Pugh B vs. C, *p* = 0.0534, Child-Pugh A vs. C, *p* = 0.0009; overall, *p* < 0.00001) (Figure 1). As for liver fibrosis markers, FIB-4 index ranged from 0.89 to 20.04 (median, 5.38), while serum hyaluronic acid ranged from 11 to 3730 ng/mL (median, 229 ng/mL). As for BIA data, extracellular water (ECW) to total body water (TBW) ratio reflecting the degree of edematous state ranged from 0.369 to 0.433 (median, 0.390). ECW to TBW ratio in healthy persons is reported to be 0.38 [29]. SMI in male ranged from 4.66–10.21 cm^2^/m^2^ (median, 7.24 cm^2^/m^2^), whereas SMI in female ranged from 3.90–7.68 cm^2^/m^2^ (median, 5.94 cm^2^/m^2^).

### 3.2. Relationship between the CONUT Score and Other Clinical Variables (Spearman’s Rank Correlation Coefficient r_s_)

Relationship between the CONUT score and other clinical variables for all cases are presented in Table 2. Significant variables with positive correlation with CONUT score were ECW to TBW ratio, total bilirubin, aspartate aminotransferase (AST), FIB-4 index, hyaluronic acid and tyrosine. Significant variables with negative correlation with CONUT score were prothrombin time (PT), platelet count, triglyceride, branched-chain amino acid (BCAA) to tyrosine ratio (BTR) and BCAA concentration. The *r_s_* values and *p* values of those parameters are listed in Table 3.

### 3.3. Univariate and Multivariate Analyses of Factors associated with CONUT Score ≥ 2 (Mild, Moderate or Severe Malnutrition)

Univariate analysis identified nine factors to be significantly associated with the presence of CONUT score ≥ 2 (*p* < 0.05): ECW to TBW ratio, total bilirubin, PT, platelet count, triglyceride, AST, BTR, FIB-4 index and hyaluronic acid (Table 4). Multivariate analysis for the seven factors (Platelet count and AST were excluded, because FIB-4 index includes platelet count and AST [30,31,32,33]) showed that ECW to TBW ratio, FIB-4 index, and BTR were significant factors linked to CONUT score ≥ 2 (Table 5). Odds ratios and 95% confidence intervals (CIs) of these factors are listed in Table 5.

### 3.4. Univariate and Multivariate Analyses of Factors Associated with CONUT Score ≥ 5 (Moderate or Severe Malnutrition)

Univariate analysis identified eight factors to be significantly associated with the presence of CONUT score ≥ 5 (*p* < 0.05): ECW to TBW ratio, total bilirubin, PT, platelet count, triglyceride, BTR, FIB-4 index and hyaluronic acid (Table 6). Multivariate analysis for the seven factors (Platelet count was excluded, because FIB-4 index includes platelet count [30,31,32,33]) showed that ECW to TBW ratio, FIB-4 index, and BTR were significant factors linked to CONUT score ≥ 5 (Table 7). Odds ratios and 95% CIs of these factors are listed in Table 7.

### 3.5. ROC Analyses for Predicting CONUT Score ≥ 2 or CONUT Score ≥ 5 in FIB-4 Index, BTR and ECW to TBW Ratio

Since FIB-4 index, BTR and ECW to TBW ratio were significant predictors linked to the presence of both CONUT score ≥ 2 and CONUT score ≥ 5, we further performed ROC analyses for those factors.

**Figure 2:**
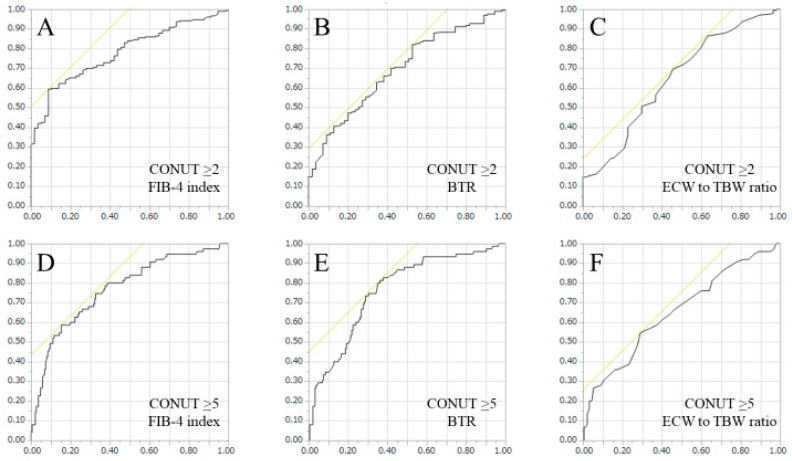
ROC curves for predicting CONUT score ≥ 2 or CONUT score ≥ 5. Horizontal axis indicates 1-specificity and vertical axis indicates sensitivity.

Corresponding AUC, optimal cutoff point, sensitivity (%) and specificity (%) of FIB-4 index, BTR and ECW to TBW ratio for predicting CONUT score ≥ 2 or CONUT score ≥ 5 were listed in Table 8. For both predicting CONUT score ≥ 2, and CONUT score ≥ 5, FIB-4 index had the highest AUC among three parameters (Table 8 and Figure 2A–F).

## 4. Discussion

To the best of our knowledge, this is one of the largest studies assessing the relationship between the CONUT score and clinical parameters in HCV-related LC patients. Although the CONUT scoring system may be established in surgically treated patients, we believe our current results are worth reporting as our data may shed some lights on the relationship between the CONUT score and clinical parameters in adult HCV-related LC patients.

In our results, the CONUT score well correlated with Child-Pugh classification and other liver functional parameters, which agree with previous reports, and the significant relationship between the CONUT score and liver functional reserve was well validated in HCV-related LC [18,34]. On the other hand, the multivariate analyses revealed that FIB-4 index, BTR and ECW to TBW ratio were independent predictors linked to the presence of both CONUT score ≥ 2 and CONUT score ≥ 5. These results denoted that FIB-4 index, BTR and ECW to TBW ratio are helpful for predicting malnutrition status in HCV-related LC patients.

It is of note that in the ROC analyses, FIB-4 index had the highest AUC for predicting both CONUT score ≥ 2 and CONUT score ≥ 5. Considering our results, in the clinical settings, in HCV-related LC patients with FIB-4 index > 5.60, some nutritional interventions may be recommended, and in HCV-related LC patients with FIB-4 index > 7.89, intensive nutritional therapies should be performed. While FIB-4 index is a well established liver fibrosis marker, this marker can also be a nutritional marker [30,31,32,33]. On the other hand, BTR reflects protein synthesis ability and its statistical significance in the multivariate analysis is not so surprising [34]. However, considering our ROC analyses, at least in HCV-related LC patients with BTR < 4.0, BCAA supplementation therapy should be considered [1,2]. ECW to TBW ratio reflects edematous status [29]. Its significance in the multivariate analysis suggest the importance of fluid management for HCV-related LC patients with malnutrition.

Advanced LC with overhydrated status can cause malnutrition and muscle wasting and lower SMI was expected to be linked to higher CONUT score, but such results were not obtained in our analysis. Overhydrated status may result in the overestimation of skeletal muscle mass and this may cause our current results [35,36].

Serum hyaluronic acid had the well correlation with the CONUT score, although it did not reach significance in the multivariate analysis. In our previous study, we reported that serum hyaluronic acid well predicts PEM in HCV-related liver disease [9]. In that study, serum hyaluronic acid level yielded the AUC (0.849) at an optimal cutoff value of 151.0  ng/mL for the presence of PEM [9]. While in this study, optimal cutoff points for the presence of CONUT score ≥ 2 and CONUT score ≥ 5 were 165 ng/mL and 295 ng/mL, respectively. Reviewing these results, PEM can develop even in patients with mild malnutrition status. In LC patients with higher CONUT score, clinicians should be aware of the presence of PEM.

Several limitations related to this study warrant mention. First, this is a retrospective observational study. Second, the study was based on a Japanese HCV-related liver disease population, and additional studies on different liver disease etiology and ethnic backgrounds are necessary to further validate and extrapolate to other backgrounds. Third, the numbers of Child-Pugh A, B or C patients were not well balanced for analysis. Fourth, dietary intake in our analyzed subjects were unclear, potentially leading to bias. However, our current results demonstrated that the CONUT score well correlates with liver function and laboratory parameters such as FIB-4 index and BTR are useful for predicting malnutrition as defined by the CONUT score.

In conclusion, the CONUT score well reflects liver functional reserve, and in particular, FIB-4 index, can be a useful marker for the presence of malnutrition in adult patients with HCV-related LC.

## Figures and Tables

**Table 1:** Controlling Nutritional Status (CONUT) score.

Variable	Normal	Mild	Moderate	Severe
Serum albumin (g/dL)	≥3.5	3.0–3.49	2.5–2.99	<2.5
Corresponding score	0	2	4	6
Total lymphocyte count (/mm^3^)	≥1600	1200–1599	800–1199	<800
Corresponding score	0	1	2	3
Total cholesterol (mg/dL)	≥180	140–179	100–139	<100
Corresponding score	0	1	2	3
Classification (sum of each score)	0 or 1	Normal nutrition status		
	2, 3 or 4	Mild malnutrition status		
	5, 6, 7 or 8	Moderate malnutrition status		
	More than 8	Severe malnutrition status		

**Table 2:** Baseline data (*n* = 264).

Variables	All Cases (*n* = 264)
Age (years)	68.0 (25.5–94.0)
Gender, Male/Female	141/123
Body Mass Index (kg/m^2^)	22.9 (13.1–34.4)
ECW to TBW Ratio	0.390 (0.369–0.433)
SMI (cm^2^/m^2^), Male	7.24 (4.66–10.21)
SMI (cm^2^/m^2^), Female	5.94 (3.90–7.68)
Upper-SMI (cm^2^/m^2^), Male	1.87 (0.80–2.82)
Upper-SMI (cm^2^/m^2^), Female	1.41 (0.83–2.03)
Lower-SMI (cm^2^/m^2^), Male	5.33 (3.86–8.19)
Lower-SMI (cm^2^/m^2^), Female	4.52 (2.93–5.88)
Child-Pugh A/B/C	198/62/4
Total Bilirubin (mg/dL)	1.0 (0.2–5.1)
Serum Albumin (g/dL)	3.7 (2.3–5.0)
Prothrombin Time (%)	78.6 (39.2–123.4)
Platelet Count (×10^4^/mm^3^)	9.9 (3.0–32.0)
eGFR (mL/min/1.73m^2^)	79.9 (6.2–164.5)
White Blood Cell (/mm^3^)	4040 (1150–9450)
Lymphocyte Count (/mm^3^)	1249 (119–3646)
Total Cholesterol (mg/dL)	149 (73–292)
Triglyceride (mg/dL)	82.5 (25–318)
CONUT Score	3 (0–10)
AST (IU/L)	43 (14–182)
ALT (IU/L)	34 (9–167)
BTR	4.05 (1.65–8.37)
BCAA (μmol/L)	423.3 (230.4–860.3)
Tyrosine (μmol/L)	107.3 (12.2–656.4)
FIB-4 Index	5.38 (0.89–20.04)
Hyaluronic Acid (ng/mL)	229 (11–3730)
Fasting Blood Glucose (mg/dL)	101 (72–403)

Data are expressed as number or median (range). ECW; extracellular water, TBW; total body water, SMI; skeletal muscle mass index, eGFR; estimated glomerular filtration rate, AST; aspartate aminotransferase, ALT; alanine aminotransferase, BTR; branched-chain amino acid (BCAA) to tyrosine ratio.

**Table 3:** Relationship between CONUT score and baseline characteristics.

	All Cases (*n* = 264)	
	*r_s_*	*p* Value
Age	0.1071	0.0823
Body Mass Index	−0.0002	0.9969
ECW to TBW Ratio	0.3470	<0.0001
SMI, Male	0.0035	0.9667
SMI, Female	0.0964	0.2888
Upper-SMI, Male	−0.0982	0.2467
Upper-SMI, Female	−0.0179	0.8439
Lower-SMI, Male	−0.0462	0.5868
Lower-SMI, Female	−0.0120	0.8955
Total Bilirubin	0.2828	<0.0001
Prothrombin Time	−0.4565	<0.0001
Platelet Count	−0.5039	<0.0001
Triglyceride	−0.2919	<0.0001
AST	0.1541	0.0122
ALT	−0.0066	0.9151
eGFR	−0.0512	0.4075
BTR	−0.4213	<0.0001
BCAA	−0.2530	<0.0001
Tyrosine	0.2888	<0.0001
FIB-4 Index	0.5465	<0.0001
Hyaluronic Acid	0.3890	<0.0001
Fasting Blood Glucose	−0.0591	0.3386

ECW; extracellular water, TBW; total body water, SMI; skeletal muscle mass index, AST; aspartate aminotransferase, ALT; alanine aminotransferase, eGFR; estimated glomerular filtration rate, BTR; branched-chain amino acid (BCAA) to tyrosine ratio.

**Table 4:** Comparison of baseline characteristics between CONUT score ≥ 2 and CONUT score < 2.

Variables	CONUT Score ≥ 2 (*n* = 207)	CONUT Score < 2 (*n* = 57)	*p* Value
Age (years)	68.0 (25.5–94.0)	66.5 (40.0–81.9)	0.1779
Gender, Male/Female	113/94	28/29	0.5490
Body Mass Index (kg/m^2^)	22.5 (13.1–34.4)	23.8 (18.2–30.3)	0.0920
ECW to TBW ratio	0.392 (0.372–0.433)	0.387 (0.369–0.400)	0.0007
SMI (cm^2^/m^2^)	6.61 (3.90–10.21)	6.57 (4.17–9.15)	0.6994
Total Bilirubin (mg/dL)	1.0 (0.2–5.1)	0.8 (0.4–2.2)	<0.0001
Prothrombin Time (%)	76.1 (39.2–123.4)	84.4 (60.5–118.7)	0.0003
Platelet Count (×10^4^/mm^3^)	8.9 (3.0–30.0)	13.4 (4.7–32.0)	<0.0001
Triglyceride (mg/dL)	77 (25–281)	98 (39–318)	0.0006
AST (IU/L)	45 (14–168)	35 (15–182)	0.0296
ALT (IU/L)	35 (9–150)	31 (9–167)	0.5416
eGFR (mL/min/1.73 m^2^)	79.7 (6.2–164.5)	81.0 (46.9–140.8)	0.8502
BTR	3.95 (1.65–8.37)	4.84 (2.56–8.31)	<0.0001
FIB-4 Index	6.39 (0.89–20.04)	3.45 (0.95–8.16)	<0.0001
Hyaluronic Acid (ng/mL)	253 (25–3730)	141 (11–1210)	<0.0001
Fasting Blood Sugar (mg/ dL)	101 (72–403)	103 (85–195)	0.6724

Data are expressed as number or median (range). ECW; extracellular water, TBW; total body water, SMI; skeletal muscle mass index, AST; aspartate aminotransferase, ALT; alanine aminotransferase, eGFR; estimated glomerular filtration rate, BTR; branched-chain amino acid to tyrosine ratio.

**Table 5:** Significant factors in the multivariate analyses for the presence of CONUT ≥ 2.

Variables	Multivariate Analysis		
	Odds Ratio	95% Confidence Interval	*p* Value
FIB-4 index	0.0011	3.274 × 10^−5^–0.0353	<0.0001
BTR	9.3126	0.9337–92.8789	0.0497
ECW to TBW ratio	0.0511	0.0033–0.7848	0.0243

When the continuous variables changed over the entire range. BTR; branched-chain amino acid to tyrosine ratio, ECW; extracellular water, TBW; total body water.

**Table 6:** Comparison of baseline characteristics between CONUT score ≥ 5 and CONUT score < 5.

Variables	CONUT Score ≥ 5 (*n* = 75)	CONUT Score < 5 (*n* = 189)	*p* Value
Age (years)	68.0 (29.4–84.6)	67.3 (25.5–94.0)	0.8123
Gender, Male/Female	37/38	104/85	0.4151
Body Mass Index (kg/m^2^)	23.1 (17.3–34.4)	22.7 (13.1–31.8)	0.0988
ECW to TBW Ratio	0.394 (0.375–0.431)	0.389 (0.369–0.433)	0.0001
Skeletal Muscle Index	6.69 (4.47–9.71)	6.58 (3.90–10.21)	0.5398
Total Bilirubin (mg/dL)	1.1 (0.4–5.1)	0.9 (0.2–2.8)	<0.0001
Prothrombin Time (%)	66.9 (39.2–104.1)	82.2 (51.5–123.4)	<0.0001
Platelet Count (×10^4^/mm^3^)	7.2 (3.0–27.8)	10.9 (3.2–32.0)	<0.0001
Triglyceride (mg/dL)	69 (25–239)	90 (25–318)	0.0066
AST (IU/L)	50 (14–139)	40 (15–182)	0.2065
ALT (IU/L)	35 (10–131)	34 (9–167)	0.7660
eGFR (mL/min/1.73 m^2^)	79.4 (23.3–146.2)	80.2 (6.2–164.5)	0.6830
BTR	3.29 (1.76–7.70)	4.44 (1.65–8.37)	<0.0001
FIB-4 index	8.40 (1.83–20.04)	4.51 (0.89–18.54)	<0.0001
Hyaluronic Acid (ng/mL)	375 (55.8–3730)	190 (11–1420)	<0.0001
Fasting Blood Sugar (mg/dL)	101 (72–233)	101 (76–403)	0.8739

Data are expressed as number or median (range). ECW; extracellular water, TBW; total body water, SMI; skeletal muscle mass index, AST; aspartate aminotransferase, ALT; alanine aminotransferase, eGFR; estimated glomerular filtration rate, BTR; branched-chain amino acid to tyrosine ratio.

**Table 7:** Significant factors in the multivariate analyses for the presence of CONUT ≥ 5.

Variables	Multivariate Analysis		
	Odds Ratio	95% Confidence Interval	*p* Value
FIB-4 Index	0.0437	0.0052–0.3180	0.0018
BTR	51.082	2.5561–1220.436	0.0095
ECW to TBW Ratio	0.0662	0.0058–0.7278	0.0266

When the continuous variables changed over the entire range. BTR; branched-chain amino acid to tyrosine ratio, ECW; extracellular water, TBW; total body water.

**Table 8:** ROC analysis for predicting CONUT score ≥ 2 and CONUT score ≥ 5.

CONUT ≥ 2	AUC	Cutoff Point	Sensitivity (%)	Specificity (%)
FIB-4 Index	0.781	5.60	59.4	91.3
BTR	0.694	5.27	82.1	47.3
ECW to TBW Ratio	0.647	0.388	69.6	54.4
CONUT ≥ 5	AUC	Cutoff point	Sensitivity (%)	Specificity (%)
FIB-4 Index	0.768	7.89	58.7	84.7
BTR	0.762	4.03	81.3	63.6
ECW to TBW Ratio	0.653	0.394	54.7	70.9

AUC; area under the receiver operating characteristic curve, BTR; branched-chain amino acid to tyrosine ratio, ECW; extracellular water, TBW; total body water.

## Abbreviations

LC: liver cirrhosis, PEM: protein energy malnutrition, SGA: subjective global assessment, CONUT: controlling nutritional status, HCV: hepatitis C virus, BIA: bioimpedance analysis, HCC: hepatocellular carcinoma, SMI: skeletal muscle mass index, ROC: receiver operating characteristics, AUC: area under the ROC curve, ECW: extracellular water, TBW: total body water, AST: aspartate aminotransferase, BTR: branched-chain amino acid (BCAA) to tyrosine ratio, PT: prothrombin time, CI; confidence interval.
